# Supplementary figures and images for: Emergent Myxobacterial Behaviors Arise from Reversal Suppression Induced by Kin Contacts
Source: mSystems. 2021 Dec 7;6(6):e00720-21. doi: 10.1128/mSystems.00720-21 (PMC8651087; doi:10.1128/mSystems.00720-21)

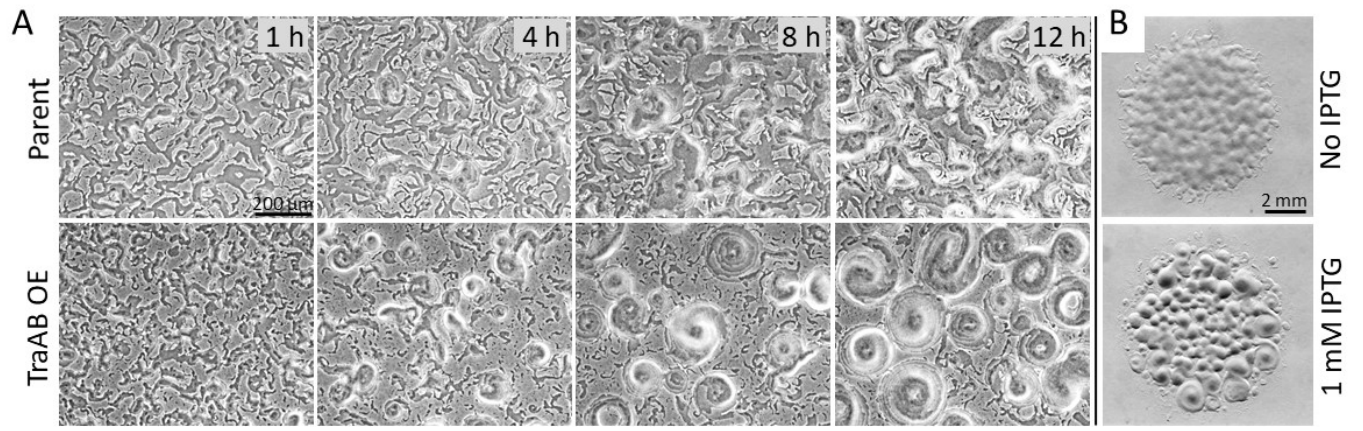

Supplement: FIG S1 [file msystems.00720-21-sf001.pdf]

A

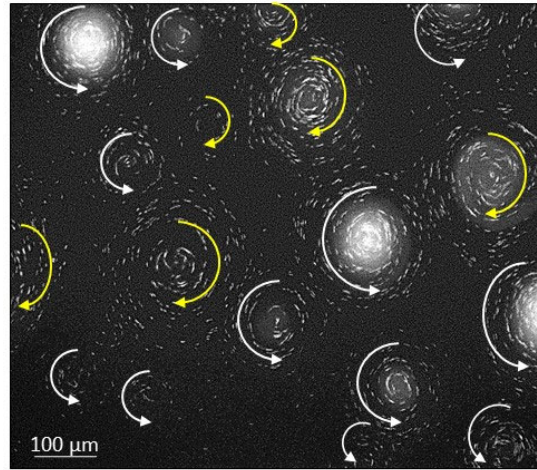

B

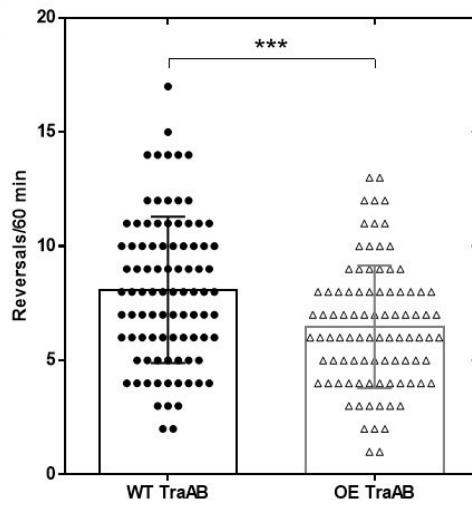

|                          | WT     | TraAB OE |
|--------------------------|--------|----------|
| Number of cells          | 91     | 91       |
| Average reversals/60 min | 8.088  | 6.473    |
| Std. Deviation           | 3.203  | 2.689    |
| P value                  | 0.0003 |          |

Supplement: FIG S2 [file msystems.00720-21-sf002.pdf]

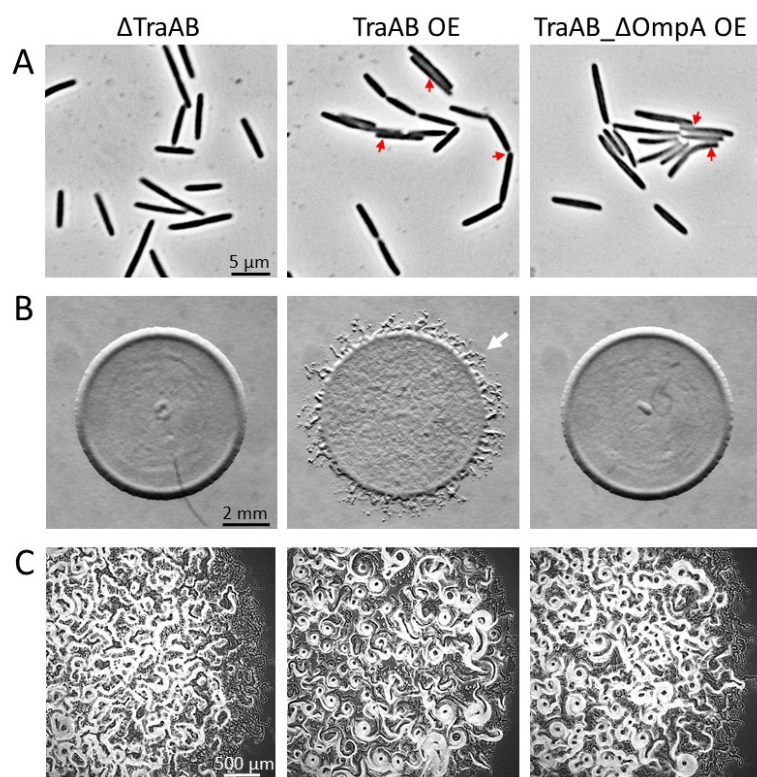

Supplement: FIG S3 [file msystems.00720-21-sf003.pdf]

**A**

IAA %

0

0.025

0.05

0.075

0.15

TraAB OE

WT

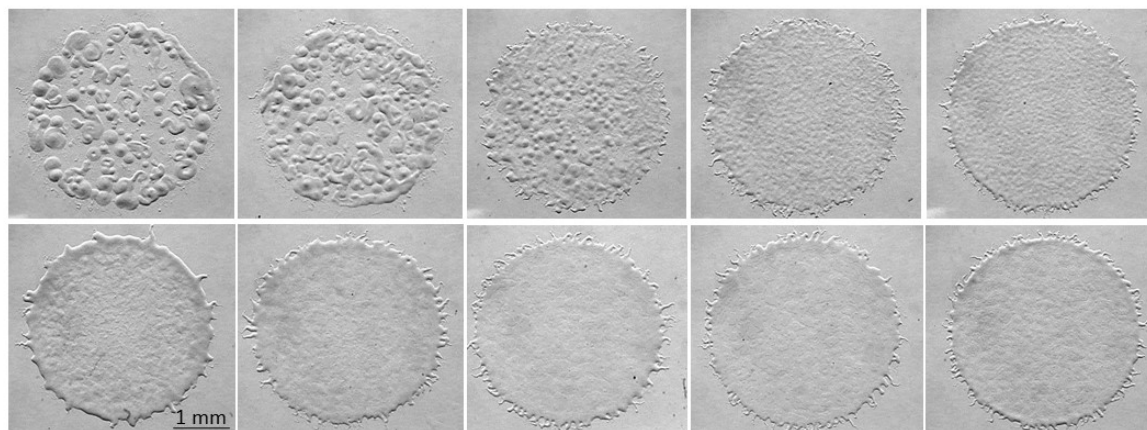**B**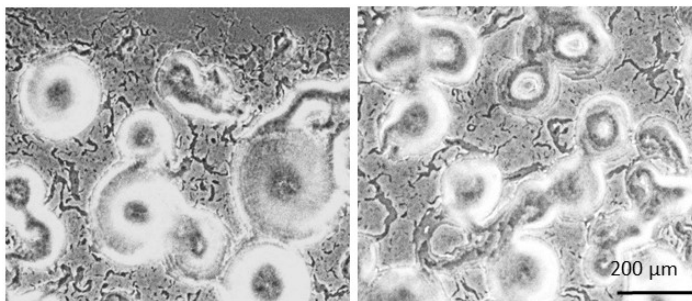

TraAB OE

 $\Delta$ Frz mutant

Supplement: FIG S4 [file msystems.00720-21-sf004.pdf]

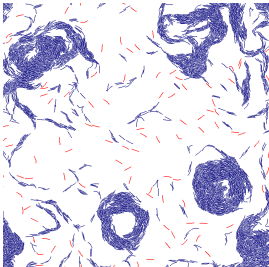

Supplement: FIG S5 [file msystems.00720-21-sf005.pdf]

# Cells

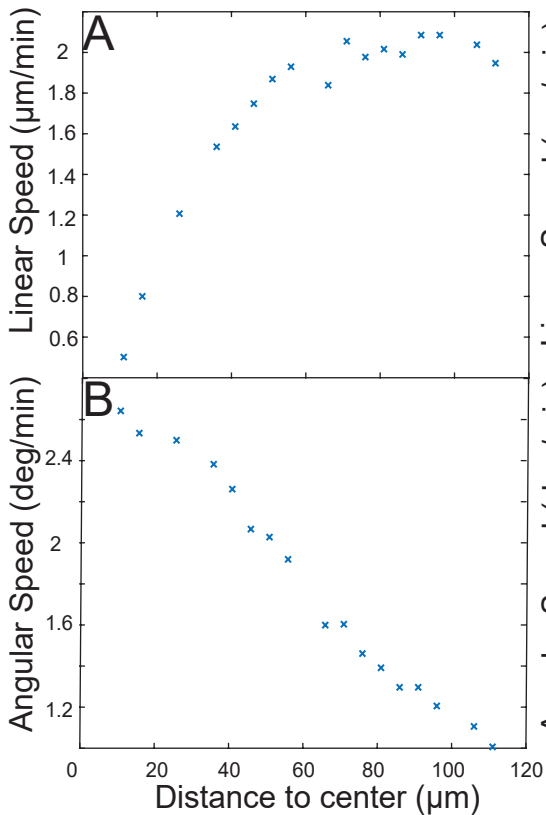

# Agents

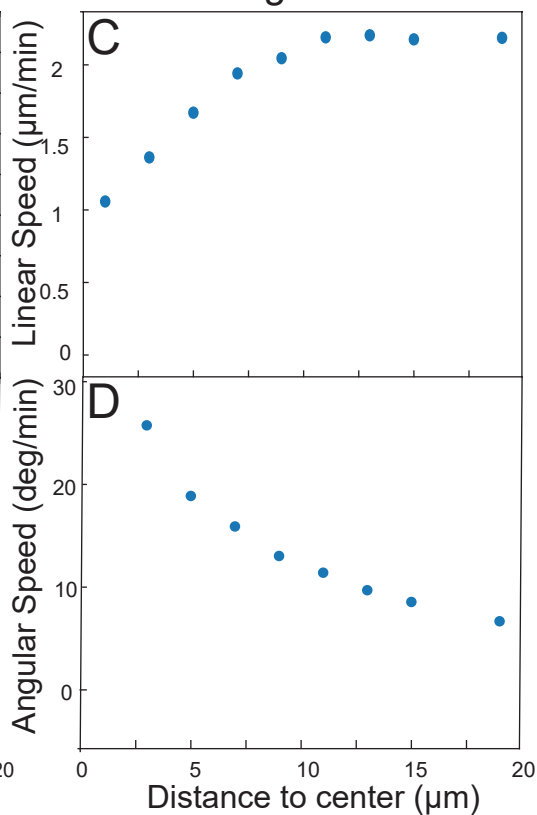

Supplement: FIG S6 [file msystems.00720-21-sf006.pdf]

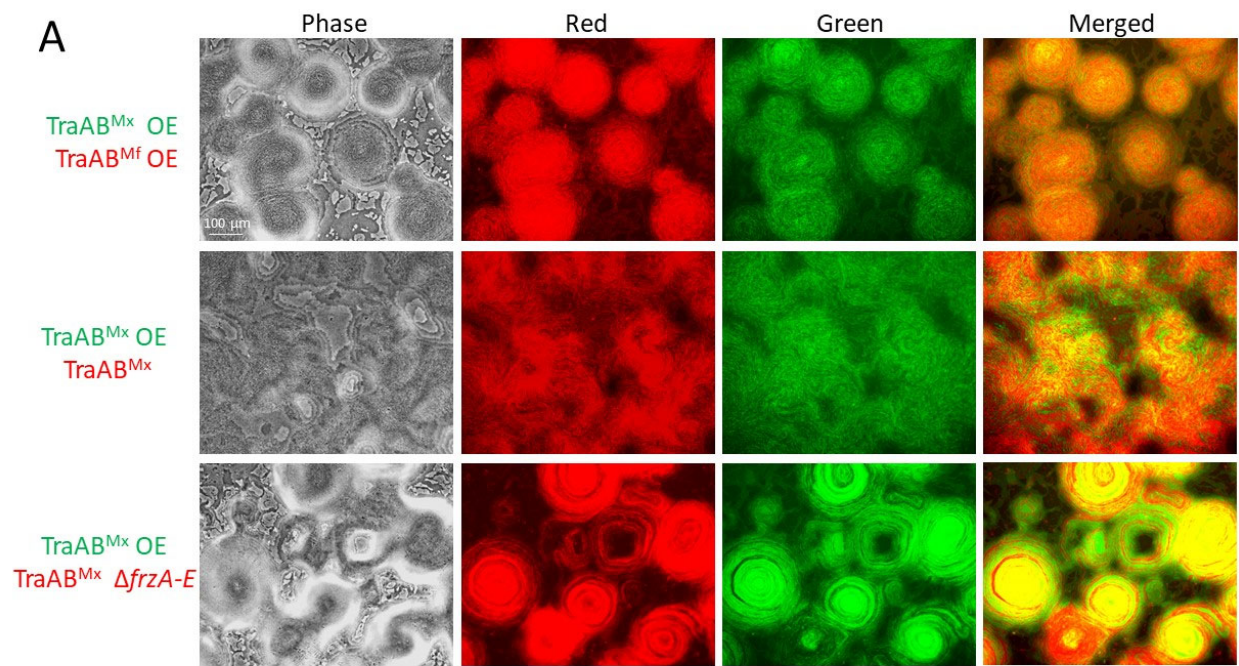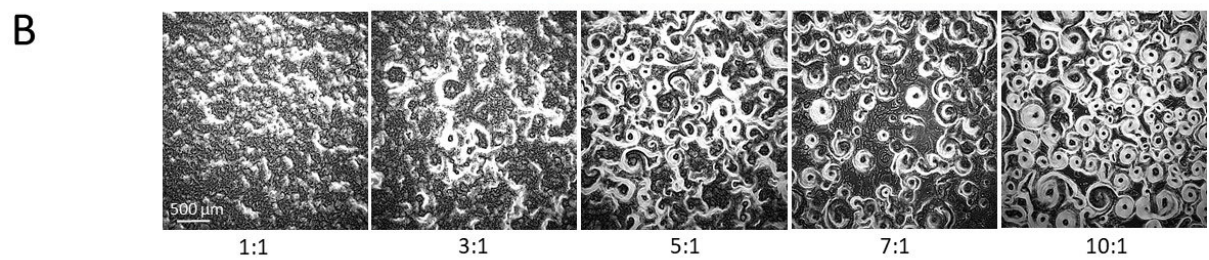

Supplement: FIG S7 [file msystems.00720-21-sf007.pdf]
